# *Chlamydia trachomatis* Infection, when Treated during Pregnancy, Is Not Associated with Preterm Birth in an Urban Safety-Net Hospital

**DOI:** 10.1155/2020/8890619

**Published:** 2020-10-05

**Authors:** Jessica Vercruysse, Samrawit Mekasha, Lisa Movilla Stropp, James Moroney, Xianbao He, Yanmei Liang, Olivera Vragovic, Eduardo Valle, Jennifer Ballard, Jeffrey Pudney, Wendy Kuohung, Robin R. Ingalls

**Affiliations:** ^1^Boston University School of Graduate Medical Sciences, Boston, MA, USA; ^2^Section of Infectious Diseases, Department of Medicine, Boston Medical Center, Boston, MA, USA; ^3^Boston University School of Medicine, Boston, MA, USA; ^4^Department of Obstetrics and Gynecology, Boston Medical Center, Boston, MA, USA; ^5^Boston University School of Public Health, Boston, MA, USA

## Abstract

Preterm birth is a major public health problem, occurring in more than half a million births per year in the United States. A number of maternal conditions have been recognized as risk factors for preterm birth, but for the majority of cases, the etiology is not completely understood. *Chlamydia trachomatis* is one of the most prevalent sexually transmitted infections in the world. However, its role in adverse pregnancy outcome in women is still debated. In order to determine if genitourinary tract infection with *C. trachomatis* during pregnancy was associated with preterm birth, we conducted a case-control study on women who delivered at Boston Medical Center, an urban “safety-net” hospital that serves a socioeconomically disadvantaged and racially diverse population. Women with known risk factors for preterm birth or immune suppression were excluded. Variables collected on enrolled subjects included demographics; diagnosis of *C. trachomatis* during or prior to pregnancy; tobacco, alcohol, and illicit substance use; gestational age; and birthweight and gender of the newborn. We also collected urine for chlamydia testing at the time of delivery and placental biopsies for nucleic acid amplification and histological studies. A total of 305 subjects were enrolled: 100 who delivered preterm and 205 who delivered full term. Among those subjects, we identified 19 cases of pregnancy-associated *C. trachomatis* infection: 6/100 preterm and 13/205 full term, a difference which was not statistically significant. Only two cases of untreated chlamydia infection were identified postpartum, and both occurred in women who delivered at term. We conclude that genitourinary tract infection with *C. trachomatis* during pregnancy, when appropriately treated, is not associated with preterm birth.

## 1. Introduction

Preterm birth (PTB) is defined by the World Health Organization (WHO) as delivery before 37 weeks gestation. Within the preterm birth category are subcategories of extremely preterm birth (less than 28 weeks), very preterm (28-32 weeks), and moderate to late preterm (32-37 weeks). It is estimated that 15 million babies are born prematurely each year, and prematurity is the leading cause of under-5 mortality worldwide [1]. In the United States and other developed countries, preterm birth is the leading cause of perinatal mortality for infants born without congenital abnormalities [2]. A review by the WHO in 2010 reported the highest rates of preterm birth in Africa, with North America a close second, and the lowest rates in Europe [3]. Statistics from the U.S. are quite alarming, as preterm birth rates continue to rise. According to the National Vital Statistics Reports from the Centers for Disease Control (CDC), overall preterm birth rates among all races rose to 10.02% in 2018, up from 9.85% in 2016 [4, 5]. Moreover, significant disparities remain in pregnancy outcomes based on race, with preterm birth rates of 14.13% among non-Hispanic black women compared to 9.09% among non-Hispanic white women in 2018 [4]. While the rates of PTB remain high, advances in neonatal medicine have improved the consequences of PTB by improving the outcome for premature infants. Unfortunately, for the infants that survive, there can be lasting disabilities, including neurologic sequelae such as cerebral palsy, mental retardation, and learning disabilities; chronic respiratory problems secondary to bronchopulmonary dysplasia; and hearing and vision impairment. Finally, women who deliver prematurely are at increased risk for cardiovascular disease later in life [6–8]. Thus, the impact on the mother, child, and their family, combined with the associated socioeconomic costs of health care, make PTB a major public health problem (reviewed in [3]).

A number of maternal factors have been linked to preterm birth, and many cases are likely mutifactorial. Infectious diseases are a known risk factor for preterm labor and have been implicated in other complications of pregnancy, such as miscarriage, stillbirth, and preeclampsia. Moreover, pregnancy has been shown to influence the immune response to a number of infections as well. Chlamydia species have long been known to be associated with abortion in ruminants. For example, *Chlamydia abortus* is one of the most important causes of abortion in sheep and goats; it can also lead to miscarriage in pregnant women exposed to infected animals [9]. However, the role of the common bacterial sexually transmitted pathogen, *C. trachomatis*, in adverse pregnancy outcome in humans is still debated. The reproductive sequelae of *C. trachomatis* infection in women of child-bearing age are well described, and even asymptomatic infection can lead to pelvic inflammatory disease, tubal infertility, and ectopic pregnancy (reviewed in [10]). Transmission of chlamydia to infants during birth, particularly in the setting of premature rupture of membranes, is a known risk factor for the development of conjunctivitis and pneumonitis [11]. Some studies have shown an association between first trimester infection with *C. trachomatis* and early miscarriage [12, 13], but the impact of infection on later pregnancy outcomes, such as preterm labor, premature rupture of membranes, and spontaneous preterm delivery is less clear. Published data on the subject is limited, and results are inconsistent, with some studies reporting an association between chlamydia infection and preterm birth [14–18] and none in others [19–22].

The standard of care in the U.S., based on recommendations from the CDC, is to screen all women under the age of 25 years for *C. trachomatis* at the first prenatal visit and retest women under the age of 25 years and those considered at high risk for infection in the third trimester to prevent postnatal complications and neonatal infections [23]. However, we believe a knowledge gap remains as to whether these recommendations are adequate, particularly in groups with high rates of both chlamydia and preterm birth. The primary objective of this study was to determine if *C. trachomatis* infection during pregnancy was associated with PTB by conducting a case-control study on women who delivered at Boston Medical Center, an urban “safety-net” hospital that serves a largely socioeconomically disadvantaged and racially diverse population.

## 2. Methods

### 2.1. Ethics Statement

All studies involving human subjects were conducted under a protocol approved by the Institutional Review Board (IRB) for the Boston University Medical Campus and in accordance with the principles expressed in the *Declaration of Helsinki*.

### 2.2. Study Design and Population

Inclusion criteria for the study were as follows. Pregnant women age 16-45 who delivered a singleton birth, regardless of parity, at Boston Medical Center between January 2013 and June 2017 were eligible for enrollment. Exclusion criteria for enrollment were HIV positive, immunosuppressive therapy, solid organ transplant, chronic hypertension, chronic kidney disease, current or prior cervical cerclage, and age less than 16 or greater than 45 at the time of enrollment. Potential participants were informed of the general outline of the investigation, i.e., assurance of the voluntary nature of the study, purpose of the study, and procedure description. The informed consent form was provided to read with unrestricted time to decide. All potential subjects were encouraged to ask questions prior to signing the consent form. Informed consent was obtained in writing by a study member in the subjects' preferred language (English, Spanish, or French). Demographic data was obtained from the medical record. Race and ethnicity were self-classified by the consenting subject. Additional data collected through the interview and/or review of the medical record was as follows: number of prenatal visits, history of spontaneous abortion, history of *C. trachomatis* infection, history of gestational diabetes, and self-reported risk behaviors including alcohol, tobacco, and substance abuse. When possible, dates of any reported chlamydia infection were confirmed in the medical record. Gestational age at delivery was based on data in the medical record, and outcome was determined to be preterm if delivery occurred before 37 weeks gestation. We did not differentiate based on cause for preterm delivery (e.g., preterm labor, premature rupture of membranes, and spontaneous preterm delivery). Preterm and full-term subjects were matched for maternal age and race. Birthweight and gender of the newborn were recorded for each subject, with the low birthweight cut-off being less than 2,500 grams. Following delivery, a wedge biopsy from the placenta was obtained prior to its being discarded, with half frozen for DNA extraction and the other half fixed in formalin for routine H&E staining. Tissue biopsies utilized in the study were all obtained within 4 hours of delivery. In order to determine if active chlamydia infection was present at the time of delivery, urine was obtained for chlamydia testing within 48 hours postpartum.

### 2.3. Detection of *C. trachomatis* in Urine

Nucleic acid transcription-mediated amplification and probe detection of *C. trachomatis* ribosomal RNA in urine samples were done using the APTIMA® Assay for *Chlamydia trachomatis* (Hologic, formerly Gen-Probe, Marlborough, MA), performed by the Boston Medical Center Microbiology Laboratory. Any positive test results were reported to the subject and her health care provider.

### 2.4. Detection of *C. trachomatis* in Placental Tissue

DNA was prepared from frozen tissue by proteinase K digestion followed by phenol-chloroform-isoamyl alcohol extraction [24]. Target sequence was amplified using the following primers: F: 5′-TGTCACAGCGGTTGCTCTAA-3′; R: 5′-CTATGCTGCAAGGAGGTAAG-3′. These primers are designed to detect the 7.5 kb cryptic plasmid of chlamydia species and yield a 317 base pair product [25].

### 2.5. Statistical Analysis

The following data were collected on the participants: demographics (age, ethnicity); history of STIs, including diagnosis of *C. trachomatis* infection during pregnancy and APTIMA® Assay for *C. trachomatis* urine test at the time of delivery; history of smoking, alcohol, and illicit substance use; and gestational age, birthweight, and gender. Data was analyzed using SAS (Version 9.4, SAS Institute, Inc., Cary, NC). Continuous variables were described by means, standard deviations, median, and interquartile ranges and categorical variables were described by frequencies and proportions. The chi-square test (for categorical data) and *t*-test and Wilcoxon rank sum test (for continuous data) were utilized to analyze differences between groups (women who deliver preterm versus those who do not). Multiple logistic regression analysis was performed to assess the association of chlamydia infection and PTB, adjusting for important clinical risk factors. Odds ratios and 95% confidence intervals were calculated to quantify effect. We controlled for potential confounders such as age, previous obstetrical history, use of cigarettes, alcohol, and drugs that were entered in the model as covariates.

## 3. Results

The primary goal of this study was to determine if preterm birth (PTB), or delivery before 37 weeks gestation, was associated with *C. trachomatis* infection during pregnancy. Subjects were enrolled in the study immediately postpartum and classified as PTB (cases) or full-term birth (FTB, controls). Sample size was calculated using an historical incidence of chlamydia infection in our obstetric patient population of 5%, based on our internal data, and an anticipated incidence of 15-20% in women with PTB, based on a review of published studies in other patient populations. Thus, a sample size of 294 with a ratio of 2 : 1 (FTB : PTB) was estimated to have 80% power to detect a difference in the history of chlamydia infection between the two groups with an alpha cut-off of 5% using the lower estimated incidence of chlamydia infection in the PTB group.

A total of 305 women were enrolled in the study, 100 PTB and 205 FTB. All enrolled subjects completed the questionnaire. Of the 305 subjects, urine samples were obtained immediately postpartum for chlamydia testing from 168 of the enrolled subjects (39 PTB and 128 FTB). The most common reasons for not collecting a urine sample were discharge from the hospital or transfer to another medical facility prior to sample collection or refusal by the subject to submit a urine sample. Placental biopsies were obtained from 210 of the enrolled subjects. The most common causes of missing biopsies were accidental discarding of tissue, delay >4 hours in obtaining tissue biopsy, or transfer of the subject to another facility for delivery. Of the 210 placental samples taken, 166 were randomly tested for *C. trachomatis* by PCR.

As shown in Table 1, both the PTB and FTB groups were matched in terms of demographics for age and race. The mean gestational age at delivery was 33.8 weeks (range, 24.2-36.6) for the PTB compared to 39.2 weeks (range, 37-41.9) for the FTB group (*p* < 0.0001). As expected, the PTB group also had significantly smaller babies with 67% meeting the criteria for low birthweight compared to 15% in the FTB group (*p* < 0.0001). Analysis of potential confounding factors demonstrated no significant difference between self-reported rates of smoking and alcohol use in the PTB versus FTB groups. In contrast, illicit substance use was associated with PTB in a statistically significant manner (21% vs. 11.2%, *p* = 0.0225).

Among our cohort of 305 subjects, only 19 *C. trachomatis* infections were identified as having occurred during pregnancy, as documented in the medical record or diagnosed by urine testing at the time of delivery. All women diagnosed with chlamydia prenatally were treated at the time of diagnosis, as per current treatment guidelines. Of these 19 cases of chlamydia, 6 delivered preterm and 13 delivered full term. Thus, we found no increased risk of PTB in women who were diagnosed with *C. trachomatis* infection during pregnancy (odds ratio of 0.9427; 95% CI: 0.3474-2.5583). Only two women were diagnosed with *C. trachomatis* infection at the time of delivery through positive urine tests obtained postpartum, and both delivered full-term babies. These cases would have otherwise gone undiagnosed as they did not report symptoms or a high-risk exposure. Of note, one subject had previously tested positive for chlamydia at the first prenatal visit and received treatment. This subject also had placental tissue test positive for *C. trachomatis* by PCR; however, the tissue appeared normal by routine H&E staining (not shown). No other placental tissue samples were determined to be positive for chlamydia DNA by PCR.

We also looked at any possible impact of more distant chlamydia infection on pregnancy outcomes. We identified 54 women who self-reported having been diagnosed with *C. trachomatis* infection at any time in the past, although this could not be confirmed in the medical record. As shown in Table 2, a past medical history of *C. trachomatis* infection was not found to be associated with an adverse outcome in terms of gestational age at delivery or birthweight. Notably, women who reported a prior history of chlamydia were younger than those who denied prior chlamydia infections, and the racial/ethnic distribution differed between the two groups as well.

## 4. Discussion

A number of factors have been linked to PTB, including maternal infection, but the association between genitourinary infection with *C. trachomatis* and PTB remains unclear. A number of observational studies have been conducted to answer this question, but results have been inconsistent, likely reflecting the heterogeneity of different populations, varying outcome definitions, and the influence of confounding factors that influence birth outcome. The unique value of our study lies with the population examined: an urban, racially diverse, and economically disadvantaged population. We were careful to match for maternal race in our study, as race has been linked to both PTB and chlamydia infections. As previously noted, the most recent U.S. statistics from the CDC demonstrated preterm birth rates of 14.13% among non-Hispanic black women compared to 9.73% among Hispanic women and 9.09% among non-Hispanic white women in 2018 [4]. Rates in Massachusetts run slightly below the national average, with overall preterm birth rates of 8.94%. Chlamydia rates are also significantly higher among Black women. For example, the overall prevalence of chlamydia in the U.S. during 2007-2012 in women aged 14-24, the population targeted for chlamydia screening, was 4.7%; the highest reported prevalence of 13.5% was among non-Hispanic black females which is seven times the prevalence among white females in the same age group [26]. Chlamydia rates also vary geographically, with the largest rates reported in the southern U.S. [27]; notably, rates in Boston, Massachusetts, are higher than the national average in specific neighborhoods [28].

We identified a total of 19 cases of chlamydia infection occurring during pregnancy in our cohort of 302 women, giving us a rate of 6.2%, and there was no statistically significant difference between the numbers of chlamydia cases during pregnancy in the PTB deliveries compared to the FTB deliveries. It should be noted that all of our subjects diagnosed with chlamydia infection prior to delivery were treated. Thus, the most we can conclude is that chlamydia-infected pregnant women who are treated appear to have no higher risk of PTB than chlamydia-uninfected women. We identified only two subjects with untreated chlamydia infection at the time of delivery, who both delivered full term. Thus, the data is insufficient to draw any conclusions as to the impact of untreated chlamydia infection on pregnancy outcomes.

There are a number of limitations to our study. First, we pooled all causes of PTB and did not differentiate between spontaneous PTB and preterm delivery for other reasons. Second, not all women provided a urine sample for chlamydia testing postpartum that could have identified untreated cases of chlamydia infection prior to delivery. The reasons for this varied. Many women were physically exhausted and unwilling to provide a urine sample immediately after delivery, but with the short hospital stay, it was not always feasible to obtain one prior to discharge. Also, given the high rates of addiction among our population, it is possible that suspicion of the health care system led some women to decline the request for providing a urine sample. Third, although the APTIMA® Assay that we used is reported to be highly sensitive and specific for both provider-collected urine and cervical swabs [29], self-collected vaginal swabs have been reported to be more sensitive than provider-collected urine using the current nucleic acid amplification tests [30]. These two factors could have led us to underestimate the number of active and untreated cases of chlamydia at the time of delivery. Finally, because of the high-risk population served at Boston Medical Center, all women are screened for chlamydia at the initial prenatal visit regardless of their age, which does differ from the CDC recommendations not to screen women over the age of 25 unless they have known high-risk behavior (more than one sex partner or partner with a known STI). Thus, unlike our BMC population, a center that stratifies screening based only on age and perceived risk might have more untreated, asymptomatic chlamydia infections among their obstetric patients.

The unique characteristics of our study population are reflected in the high reported rates of smoking and substance use during pregnancy. Surprisingly, we found no significant association between smoking and PTB in our cohort, which has been reported in other studies. For example, the CDC reported in 2010 that 11.5% of all births had exposure to prenatal smoking, which was significantly associated with preterm deliveries and term low birthweight deliveries [31]. Considering that smoking rates overall are decreasing, and self-reported smoking generally underestimates the rates, our cohort had considerably higher rates of smoking than that described in other studies, with an overall rate of 23% that did not differ between the PTB and FTB groups. We did find a statistically significant difference between illicit substance use in the PTB versus FTB groups, with women who self-reported illicit substance use more likely to deliver preterm than those who did not. We did not stratify the subjects who reported substance use by type of exposure (opioid, cocaine, polysubstance use, etc.) nor did we collect data on whether they were engaged in treatment for opioid use disorder and, if so, with what.

In summary, chlamydia infections that occur during pregnancy do not appear to impact the likelihood PTB provided they are treated appropriately. We cannot quantify the impact of untreated chlamydia infection on pregnancy outcome due to the low numbers of undiagnosed/untreated chlamydia infections that we identified in our study. The observation that illicit substance use is more common in women who deliver preterm, while perhaps not surprising, should be a call to further investigate the potential biological, social, and socioeconomic factors associated with substance use disorder that could contribute to adverse birth outcomes in order to improve the health of women and infants, particularly in a vulnerable population such as ours.

## Figures and Tables

**Table 1:** Characteristics associated with preterm and full-term births.

	Preterm birth (PTB) (*n* = 100)	Full-term birth (FTB) (*n* = 205)	*p* value
Age (mean ± STDEV)	28.8 ± 5.9	28.1 ± 5.9	0.37^∗^
Race *n* (%)			
(i) White (ii) Black (iii) Hispanic (iv) Other	22 (22.0%) 48 (48.0%) 28 (28.0%) 2 (2%)	42 (20.5%) 101 (49.3%) 55 (26.8%) 7 (3.4%)	0.90^∗∗^
Number of prenatal visits mean ± STDEV (*n* = 251)	5.6 ± 4.3	8.8 ± 3.6	<0.0001^∗^
Gestational age at delivery (range, weeks) mean ± STDEV	33.7 ± 3.3 (24.2-36.6)	39.5 ± 1.2 (37-41.9)	<0.0001^∗^^,1^
Low birthweight *n* (%)	67 (67.7%)	30 (14.6%)	<0.0001^∗∗^
Substance abuse *n* (%)	21 (21.0%)	23 (11.2%)	0.0225^∗∗^
Tobacco risk *n* (%)	26 (26.0%)	43 (21.0%)	0.32^∗∗^
Alcohol risk *n* (%) (*n* = 300)	3 (3.0%)	2 (1.0%)	0.34^#^
History of chlamydia during pregnancy *n* (%)	6 (6%)	13 (6.3%)	0.83^∗^

^∗^Two-sample *t*-test; ^∗∗^chi-square test; ^#^Fisher's exact test. ^1^After adjusting for gestational age at delivery, the *p* value was 0.85.

**Table 2:** Characteristics of women with and without prior history of chlamydia infection.

	Any history of chlamydia+ (*n* = 54)	Any history of chlamydia− (*n* = 251)	*p* value
Age mean ± STDEV	25.6 ± 5.2	29.0 ± 5.8	0.0001^∗^
Race n (%) (*n* = 305)			
(i) White (ii) Black (iii) Hispanic (iv) Other	5 (9.3%) 41 (75.9%) 8 (14.8%) 0 (0%)	59 (23.5%) 108 (43.0%) 75 (29.9%) 9 (3.6%)	<0.0001^#^
History of spontaneous abortion *n* (%) (*n* = 305)	15 (27.8%)	64 (25.5%)	0.73
Substance abuse (yes) *n* (%)	11 (20.4%)	33 (13.2%)	0.17^∗∗^
Tobacco risk (yes) *n* (%)	16 (29.6%)	53 (21.1%)	0.17^∗∗^
Alcohol risk (yes) *n* (%) (*n* = 300)	2 (3.7%)	3 (1.2%)	0.22^#^
Gestational age at delivery (range, weeks) mean ± STDEV (*n* = 305)	38.0 ± 3.0 (24.2-41.9)	37.5 ± 3.5 (24.3-41.6)	0.27^∗^
Low birth weight *n* (%) (yes) (*n* = 300)	16 (30.2%)	81 (32.8%)	0.71^∗∗^

^∗^Two-sample *t*-test; ^∗∗^chi-square test; ^#^Fisher's exact test.

## Data Availability

Additional data available on request from the corresponding author.
